# Supine to standing Cobb angle change in idiopathic scoliosis: the effect of endplate pre-selection

**DOI:** 10.1186/1748-7161-9-16

**Published:** 2014-10-08

**Authors:** Bethany E Keenan, Maree T Izatt, Geoffrey N Askin, Robert D Labrom, Mark J Pearcy, Clayton J Adam

**Affiliations:** 1 Paediatric Spine Research Group, Institute of Health and Biomedical Innovation, Queensland University of Technology and Mater Health Services, Brisbane, Queensland 4101, Australia

## Abstract

**Background:**

Supine imaging modalities provide valuable 3D information on scoliotic anatomy, but the altered spine geometry between the supine and standing positions affects the Cobb angle measurement. Previous studies report a mean 7°-10° Cobb angle increase from supine to standing, but none have reported the effect of endplate pre-selection or whether other parameters affect this Cobb angle difference.

**Methods:**

Cobb angles from existing coronal radiographs were compared to those on existing low-dose CT scans taken within three months of the reference radiograph for a group of females with adolescent idiopathic scoliosis. Reformatted coronal CT images were used to measure supine Cobb angles with and without endplate pre-selection (end-plates selected from the radiographs) by two observers on three separate occasions. Inter and intra-observer measurement variability were assessed. Multi-linear regression was used to investigate whether there was a relationship between supine to standing Cobb angle change and eight variables: patient age, mass, standing Cobb angle, Risser sign, ligament laxity, Lenke type, fulcrum flexibility and time delay between radiograph and CT scan.

**Results:**

Fifty-two patients with right thoracic Lenke Type 1 curves and mean age 14.6 years (SD 1.8) were included. The mean Cobb angle on standing radiographs was 51.9° (SD 6.7). The mean Cobb angle on supine CT images without pre-selection of endplates was 41.1° (SD 6.4). The mean Cobb angle on supine CT images with endplate pre-selection was 40.5° (SD 6.6). Pre-selecting vertebral endplates increased the mean Cobb change by 0.6° (SD 2.3, range -9° to 6°). When free to do so, observers chose different levels for the end vertebrae in 39% of cases. Multi-linear regression revealed a statistically significant relationship between supine to standing Cobb change and fulcrum flexibility (p = 0.001), age (p = 0.027) and standing Cobb angle (p < 0.001). The 95% confidence intervals for intra-observer and inter-observer measurement variability were 3.1° and 3.6°, respectively.

**Conclusions:**

Pre-selecting vertebral endplates causes minor changes to the mean supine to standing Cobb change. There is a statistically significant relationship between supine to standing Cobb change and fulcrum flexibility such that this difference can be considered a potential alternative measure of spinal flexibility.

## Introduction

Adolescent Idiopathic Scoliosis (AIS) is a complex, three dimensional deformity of the spinal column and trunk. It is characterized by an abnormal lateral curvature of the spinal column, axial rotation of the vertebrae, and a loss of lordosis and kyphosis in the sagittal plane. The progression and treatment of AIS is monitored clinically with coronal plane standing radiographs, using a measure of curve severity known as the Cobb angle [1]. In certain cases (typically to assist with diagnosis or for pre-operative planning), supine imaging modalities such as CT or MRI are also used. In these instances, it is important to know the difference in scoliosis curve geometry between supine and standing positions. Prior studies have reported major Cobb angles 7-10° [2-5] smaller in the supine position than in standing due to changes in gravitational loading direction.

To the best of our knowledge, only two studies to date have directly measured the difference in Cobb angle between supine and standing positions [3,5]. Torell et al. [5] reported a mean 9° Cobb difference for a group of 287 female patients (aged 10–17 years, with mean supine Cobb of 30.6° and 39.4° in standing). Similarly, Lee et al. [3] found a 10° Cobb difference for a group of 70 patients (40 female and 30 male, aged 10–18 years with mean supine Cobb angle of 48° and 58° in standing). Although not directly comparing supine and standing positions, two other studies have measured the Cobb change between supine axially loaded and non-loaded cases using MRI [2,4]. Adam et al. [2] found a mean Cobb difference of 7° for a group of 10 patients and Wessberg et al. [4] reported an 8° Cobb change for 30 patients.

However, both the Wessberg and Lee studies used the vertebral endplates selected on the standing radiograph to measure the Cobb angles on supine MRI images. Torell et al. [5] state that the radiographs were measured using routine techniques but does not state whether the end-vertebrae were pre-selected for the supine Cobb angles or not. Adam et al. [2] measured Cobb angles for ten patients on supine MRI without endplate pre-selection. Given that endplate pre-selection has been shown to affect Cobb measurement variability [6] and that the change in spinal configuration from supine to standing postures could result in a shift in the end vertebrae of a scoliotic curve, the primary aim of this study was to examine the effect of endplate pre-selection on the difference in Cobb angle and number of levels comprising the major curve between supine versus standing. A second aim of this study was to identify which (if any) patient characteristics were correlated with supine versus standing Cobb difference.

## Materials and methods

A series of existing low-dose CT scans taken between 2002 and 2008 for a group of female, Lenke type 1 AIS patients were used retrospectively to measure scoliosis Cobb angles. A single low-dose CT scan was part of the pre-operative clinical assessment process at the time, for those patients who were scheduled to receive a thoracoscopic anterior spinal fusion to assist with safer screw sizing and positioning [7]. As the CT data was from a clinical dataset, ethics clearance was not required at this time and subsequent analysis of the existing CT scans was considered a clinical audit and therefore exempt from requiring ethical review.

Three different CT scanners were used over the six year period of the study; (i) a 64 slice GE Lightspeed Plus (GE Healthcare, Chalfont St. Giles, UK) (ii) a 64-slice Philips Brilliance (Philips Healthcare, Andover, USA) and (iii) a 64 slice GE Lightspeed VCT (GE Healthcare, Chalfont St. Giles, UK). The scan coverage in each case was from C7 to S1. Dose reports were commissioned for all three scanners, and the highest estimated radiation dose of 3.0 mSv occurred with the oldest scanner (GE Lightspeed Plus), with uncertainties due to the dose model in the order of ±20% [8]. By comparison, the combined dose for a postero-anterior (PA) and lateral standing radiographs is in the order of 1.0 mSv, and the annual background radiation in Queensland, Australia is approximately 2.0 mSv per annum [8,9]. Estimated doses for the newer 64 slice scanners were substantially lower (in the order of 2 mSv). Subjects were in a supine position with the upper limbs positioned over the head during CT scanning. The time period between the CT scan and plain radiograph did not exceed 3 months.

Patients’ ages ranged from 11 to 18 years and Cobb angles ranged from 40-70° with none having a leg length discrepancy of more than 1 cm. All patients received PA and lateral standing radiographs and a fulcrum bending radiograph as part of routine clinical assessment prior to scoliosis correction surgery [10,11]. The Cobb angle (using the most tilted vertebrae above and below the apex of the curve), fulcrum flexibility [10], Risser sign [12] (classification system to measure skeletal maturity) and any leg length discrepancy were found from the patient’s clinical records. Spinal flexibility in idiopathic scoliosis is clinically assessed using a pre-operative fulcrum bending radiograph, whereby the patient is required to lie laterally over a cylindrical bolster positioned at the curve apex. Equation 1 is used to define Fulcrum Flexibility [10].

(1) $$\begin{array}{c} \text{Fulcrum}\;\text{Flexibility}=\frac{Pre‒\text{operative}\;\text{Cobb}‒\text{Fulcrum}\;\text{bending}\;\text{Cobb}}{Pre‒\text{operative}\;\text{Cobb}}\\ \;\times 100\% \end{array}$$

**Figure 1:**
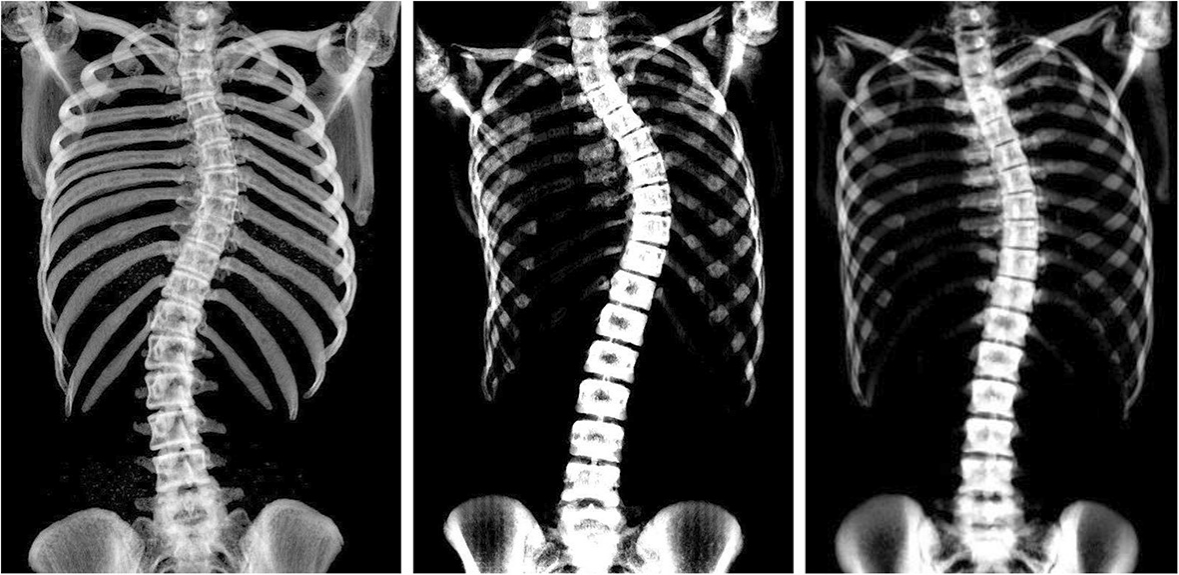
Reformatted coronal CT images of idiopathic scoliosis patients, used for supine Cobb angle measurements.

Experienced clinicians measured the Cobb angle on the patients’ standing radiographs as part of their routine clinic assessment at the hospital. Two observers in the current study re-measured each radiograph to verify these clinical standing Cobb measures. In cases where both observers measured Cobb angles more than 5° different (the accepted intra-observer variability error) to those recorded in the clinical charts, a clinician was asked to blindly re-measure the patient radiograph to ensure measurement or recording error had not occurred in the records. Observer 1 was a post-graduate student with a degree in medical engineering and 2 years of clinical Cobb angle measuring experience. Observer 2 was a senior research assistant with 14 years’ experience in clinical Cobb angle measurement.In order to measure Cobb angles from the supine CT scans, the ImageJ software (v. 1.45) National Institutes of Health, USA) was used to create reformatted coronal plane images from the axial CT slices. Combining the reformatted coronal slices into a single image was performed using the z-project function in ImageJ. Figure 1 shows example of the reformatted coronal images obtained using this technique. A hardcopy of each reformatted coronal CT image was printed onto an A4 sheet of paper at a scale of approximately 60%, to allow each observer to measure the Cobb angle using the standard Cobb method. To ensure the image was not distorted during scaling the aspect ratio of each image was locked.

The two observers manually measured the Cobb angle on the reformatted coronal CT images for each patient using two methods: (1) the observer was permitted to select the endplates of the major curve and (2) the observer was provided with the pre-selected endplates from the standing radiograph and used these same levels for the supine CT Cobb measurement. Both observers were blinded to patient identity and patient order was randomized. Observer 1 repeated the measurements for both methods 5 weeks later to determine the intra-observer variability. The repeated measurements were performed on fresh printouts with no previous markings.

To avoid confusion in the presentation of results, the following terminology is used: supine to standing *Cobb change* refers to the increase in Cobb angle measurement from supine to standing e.g. if a supine scan measures 50° Cobb angle and a standing radiograph for the same subject measures 61°, then the supine to standing Cobb change for that patient is 11°. The term *difference* is only used to refer to inter and intra-observer measurement differences. For example, if Observer 1 measures a Cobb change of 8° and Observer 2 measures the same patient’s Cobb change to be 10° then the inter-observer difference (in supine to standing Cobb change) for this patient is 2°.

### Statistical analysis

Multi-linear regression was used to investigate whether there was a relationship between supine to standing Cobb change and patient characteristics using the statistics package SPSS (v. 21, IBM, USA). The dependent variable was assigned as supine to standing Cobb change (in degrees) and the eight independent variables explored were; patient age (yrs), mass (kg), standing Cobb angle (°), Risser Sign (0 – 5), ligament laxity (0 – 5), Lenke type 1 lumbar modifier (i.e. A, B or C), fulcrum flexibility (%) and time delay between standing radiograph and CT scan (months).

## Results

### Demographics

The patient demographics for each of the 52 female AIS patients can be seen in Table 1. Individual Cobb angle measures recorded for each patient’s supine (with and without endplate pre-selection) and standing image can be found in the Additional file 1. The mean age of the group was 14.6 years (SD 1.8) and all curves were right-sided major thoracic Lenke Type 1 with 30 patients classified as lumbar spine modifier A, 13 as lumbar modifier B and 9 as lumbar modifier C. The mean time interval between the standing plain radiograph and the supine CT scan was 1.0 (SD 0.5) months.

**Table 1:** Patient demographics for the 52 female idiopathic scoliosis patients, divided into nominal age groups

**Nominal age at CT scan (yrs)**	**Mean age (yrs)**	**Number of patients**	**Mean mass (kg)**	**Risser sign (0–5)**	**Ligament laxity (0–5)**	**Mean supine Cobb angle (°) (range)**	**Mean standing Cobb angle (°) (range)**	**Mean change in Cobb (°)**	**Lenke type 1**		
									**A**	**B**	**C**
All	14.6	52	52	0-5	0-5	42 (28–57)	52 (40–68)	11	30	13	9
11	11.5	2	44	0	0-2	42 (39–45)	50 (48–52)	8	0	2	0
12	12.4	6	44	0-3	0-3	44 (39–48)	56 (47–64)	12	6	0	0
13	13.5	15	50	0-4	0-4	46 (35–57)	54 (40–63)	8	9	2	4
14	14.5	11	60	0-5	0-4	42 (31–55)	51(42–62)	9	4	6	1
15	15.3	7	53	0-5	0-5	40 (34–51)	51(44–68)	11	3	1	3
16	16.5	3	54	3-5	0-2	36 (30–40)	47 (42–50)	11	2	0	1
17	17.5	5	53	4-5	0-3	36 (28–43)	48 (38–58)	12	4	1	0
18	18.1	3	52	5	0-2	40 (32–49)	52 (42–58)	12	2	1	0

### Effect of endplate pre-selection on Cobb angle and Cobb change

**Figure 2:**
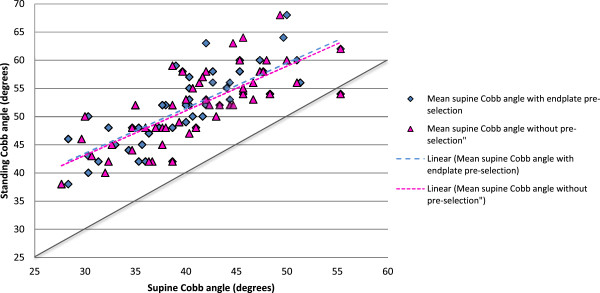
**Standing Cobb angles versus mean supine Cobb angles, with and without pre-selection.** The solid diagonal line indicates a 1:1 correspondence between supine and standing Cobb angles. The dashed lines are almost identical and show that the effect of endplate pre-selection is negligible.

The mean thoracic Cobb angle measured on standing radiographs was 51.9° (SD 6.7). The mean thoracic Cobb angle on supine CT images *without* endplate pre-selection was 41.1° (SD 6.4). The mean thoracic Cobb angle on supine CT images *with* endplate pre-selection was 40.5° (SD 6.6). Figure 2 shows a scatter plot of standing versus supine Cobb angles for the entire patient cohort with and without endplate pre-selection. As expected, the two regression lines are almost identical for the supine Cobb angles, as the difference between pre-selecting and not pre-selecting endplates was negligible.

**Figure 3:**
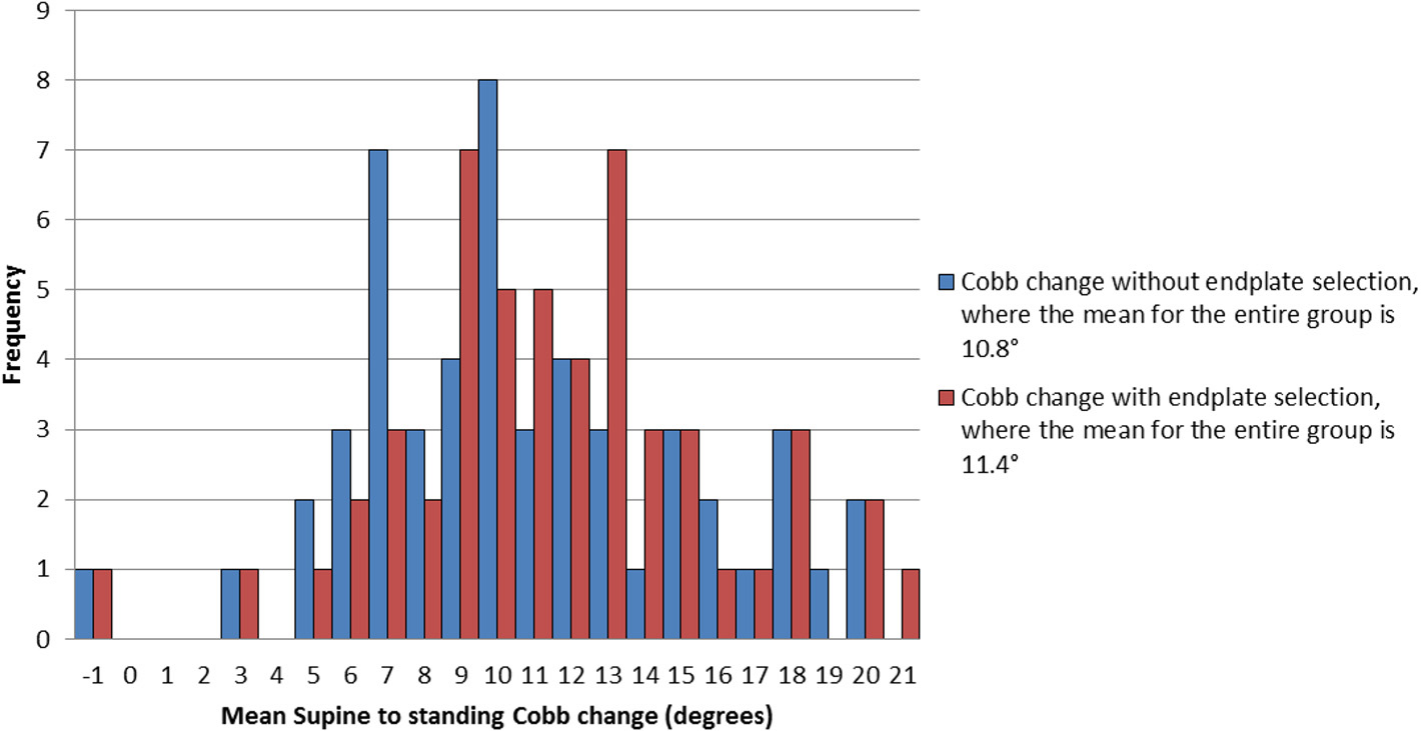
Distribution of mean supine - standing Cobb change with (red) and without (blue) pre-selecting vertebral levels.

For the entire patient cohort, (N = 52), when Cobb angles were measured on supine CT *without* endplate pre-selection, the mean supine to standing Cobb change was 10.8° (SD 4.8). When Cobb angles were measured using the pre-selected levels from the standing radiograph the mean supine to standing Cobb change was 11.4° (SD 4.5). Pre-selecting vertebral endplates therefore increased the mean Cobb change by only 0.6° (SD 2.3, range -9 to 6) compared to the measurements without pre-selection. Figure 3 shows the range of supine to standing Cobb changes for the entire group and the effect of endplate selection on Cobb change.

### Distribution of Cobb changes

Whilst the mean supine to standing Cobb change for the patient group was 10.8°, there were individual cases (see Figure 3) where patients had supine to standing Cobb changes of up to 20°. Of the 52 patients, 29 (56%) had Cobb changes ≤10°. In 11 patients (21%) the Cobb changes ranged from 10-14° and in 12 patients (23%) the Cobb changes were in the range 15-20°. One patient in the study had a mean supine to standing Cobb change of -1°, implying that the supine Cobb angle measured on the CT scan was essentially the same as that of the standing radiograph.

### Variation in selected end vertebrae

Each patient’s supine Cobb angle was measured three times, such that observers chose upper and lower end vertebrae for 156 supine Cobb angles. In 75 (48%) of the 156 supine Cobb angles measured, the upper and lower endplates chosen by the observers on the supine CT scan were identical to those measured clinically on the standing radiograph. For the remaining 81 (52%) of the 156 supine Cobb angles measured, the endplates chosen by the observer were different to those measured on the standing radiograph. For these 81 measurements, the average supine to standing Cobb change was 11.1° (SD 4.8), whereas pre-selecting vertebral levels caused the mean supine to standing Cobb change to be 11.9° (SD 4.3). Therefore the difference in mean Cobb change (between non-preselected and pre-selected endplates) was 0.8° (SD 2.8, range -9 to 6). This is slightly higher than the 0.6° difference in Cobb change between non-preselected and pre-selected measurements given above for the entire patient group, but still not significant when compared to the generally used 5° threshold denoting a clinically relevant Cobb difference.

### Number of vertebrae in major curve

**Figure 4:**
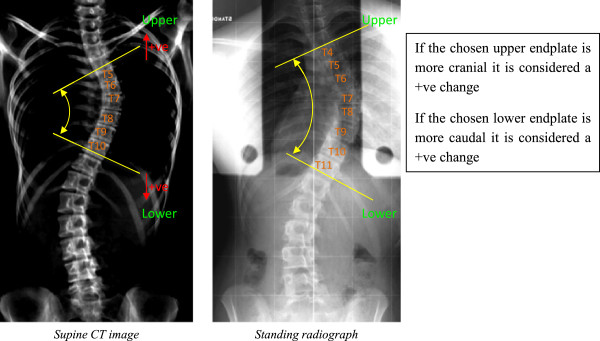
The number of vertebrae included in the major curve can change between supine and standing.

**Figure 5:**
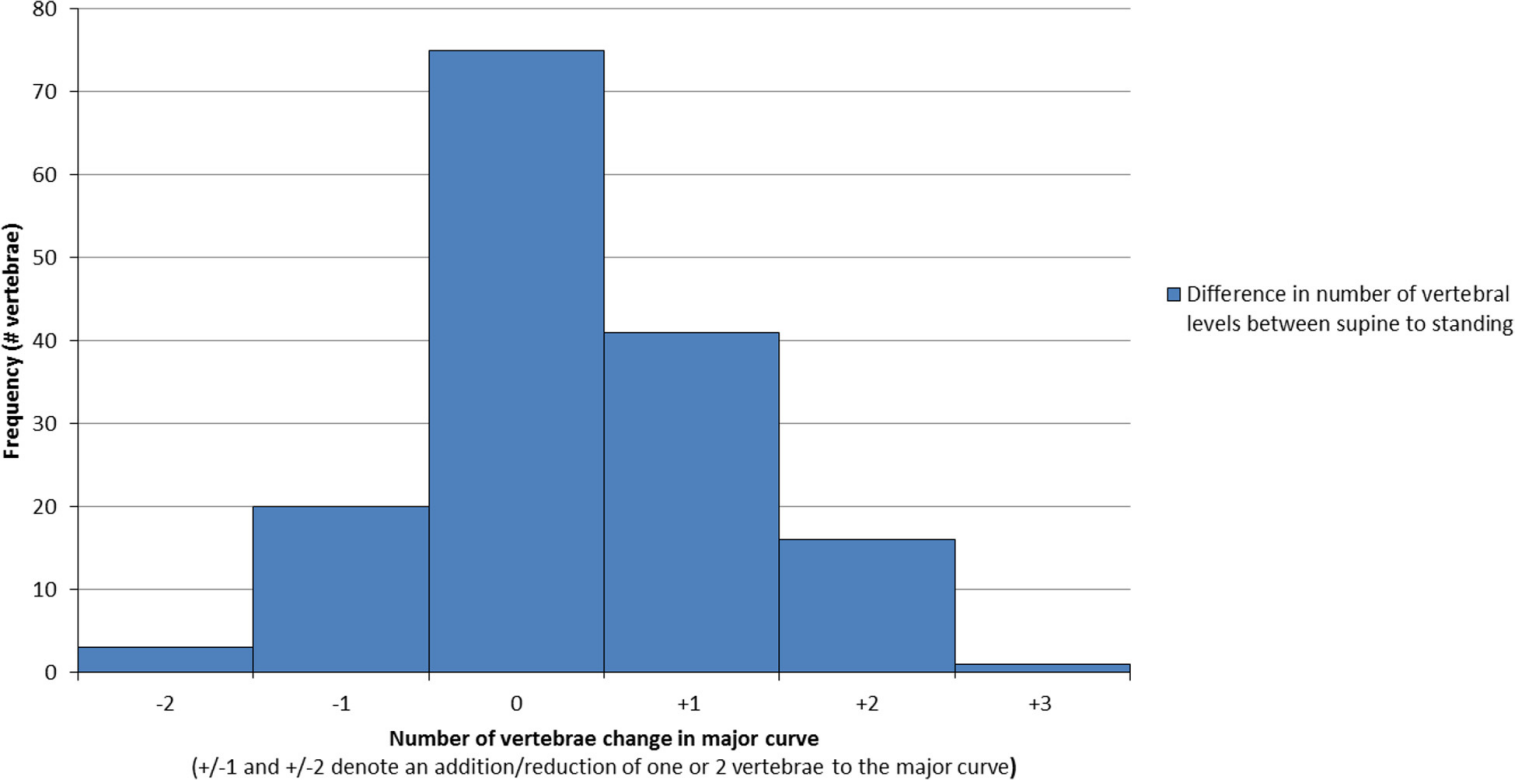
Changes in the number of vertebrae between the supine CT scan and standing radiograph.

When considering how the number of vertebrae included in the major curve changed between supine and standing positions, a convention was adopted in which addition of a vertebra at either extent of the major curve between supine and standing positions was denoted as a positive (+) change, as shown in Figure 4. Using this convention, Figure 5 shows a histogram of the vertebral level changes between the supine CT scan and the radiograph (when the observers selected levels). As already described, in almost half the cases the upper and lower endplates of the major curve did not change between supine and standing positions (75 out of the 156 Cobb measures taken). However when there were changes, these tended to increase the number of vertebrae in the major curve (+1 values in Figure 5) rather than to decrease the number of vertebrae (-1 values), with 41 cases (26%) having an additional vertebra in the major curve and 20 cases (13%) displaying a reduction of one vertebra. In very few cases there were changes of two or three vertebral levels in the major curve between supine and standing (+/-2 and +3 values in Figure 5) with only 17 cases (11%) showing an increase of two or three vertebrae and 3 cases (2%) showing a reduction of two vertebrae. These results indicate that the number of vertebrae in the scoliotic curve tends to increase from supine to standing, but the effect is marginal.

### Statistical analysis – predictors of Cobb change

Multi linear regression (shown in Table 2) revealed a statistically significant relationship between the mean supine to standing Cobb change and three of the candidate independent variables: fulcrum flexibility (p = 0.001), age (p = 0.027) and standing Cobb angle (p < 0.001) with an R-squared value 38% (for all three variables combined). Patient mass, Risser sign, ligament laxity, Lenke lumbar modifier and the time interval between the CT scan and radiograph were not found to be statistically significant.

**Table 2:** Multi-linear regression results using SPSS: where the dependent variable was Supine to Standing Cobb change

**Model**	**Unstandardized coefficients**		**Significance**	
	**B**	**Std. Error**		
(Intercept/Constant)	-29.039	8.027	.001	
Standing Cobb from X-ray (°)	.408	.084	.000	Independent variables
Age (years)	.686	.301	.027	
Fulcrum flexibility (%)	.053	.044	.001	

Note: Unstandardized coefficients refer to the change in predicted Y for one unit change in X. B coefficients are the values for the regression equation for predicting the dependent variable from the independent variable. Std. Error is the standard error associated with the coefficients.

### Intra-observer variability

Intra-observer variability was assessed by analyzing the absolute supine to standing Cobb change (α) measurements by the same observer,

$$\Delta \alpha =\left|\alpha_{n}-\alpha_{n+1}\right|$$

**Figure 6:**
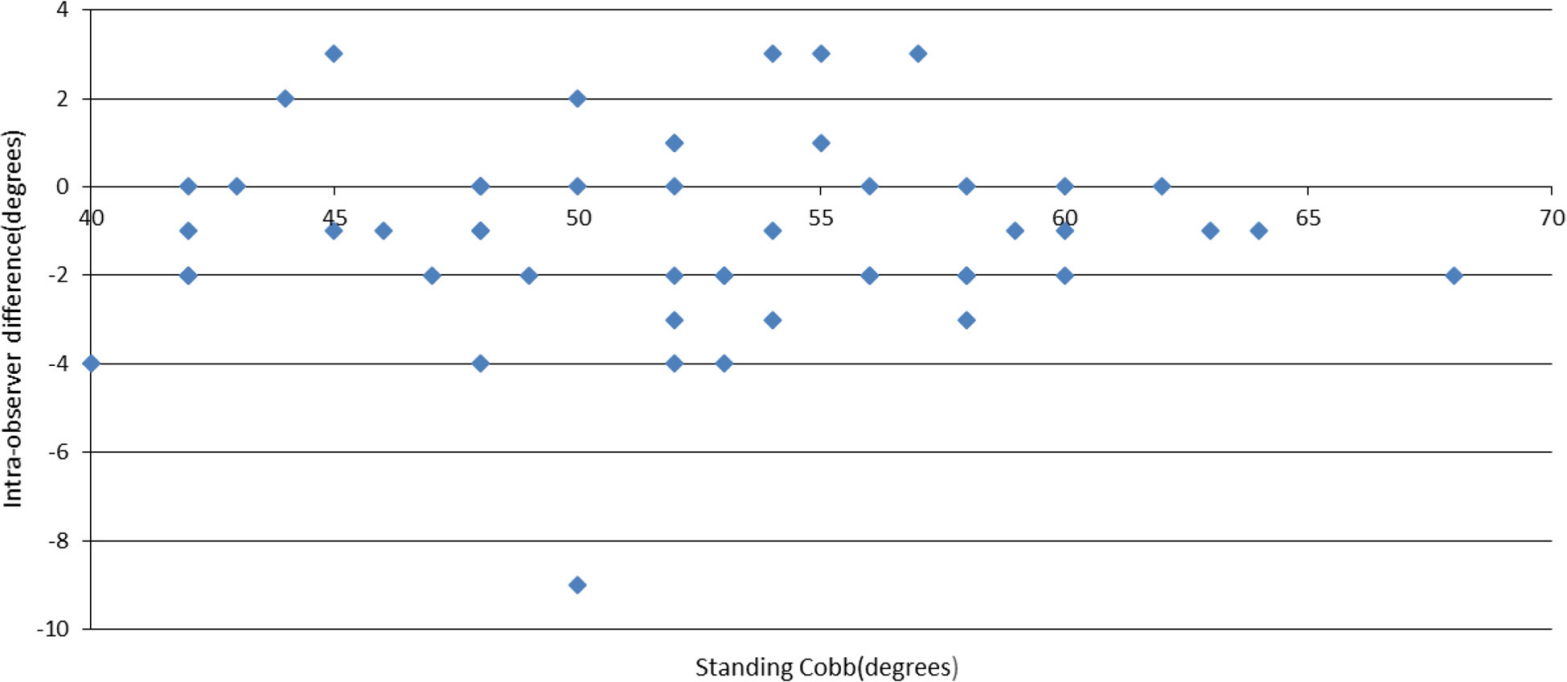
Intra-observer difference in supine to standing Cobb angle change versus standing Cobb angle.

Where *n* and *n* + 1 are successive measurements. Figure 6 shows a scatter plot of signed measurement difference (α_*n*_ - α_*n* + 1_) *versus* standing Cobb measure. The mean signed intra-observer difference was -0.9°, which is not significantly different from zero and suggests that no order bias existed between the first and second Cobb change measurements in a pair. Mean absolute (unsigned) intra-observer difference was 1.8°, the standard deviation (SD) of the difference was 1.6° and the 95% confidence interval (CI) (1.96 × SD) was ± 3.1°. There was no statistically significant correlation between intra- observer variability and standing Cobb measurements. i.e. patients with larger Cobb angles did not tend to have greater intra-observer measurement variability.

### Inter-observer variability

Inter-observer variability for measurement of supine to standing Cobb change on reformatted coronal CT images was assessed using the approach described by Bland and Altman [13,14]. The inter-observer difference (α) was calculated as:

$$\Delta \alpha =\left|\alpha_{n}-\alpha_{m}\right|$$

**Figure 7:**
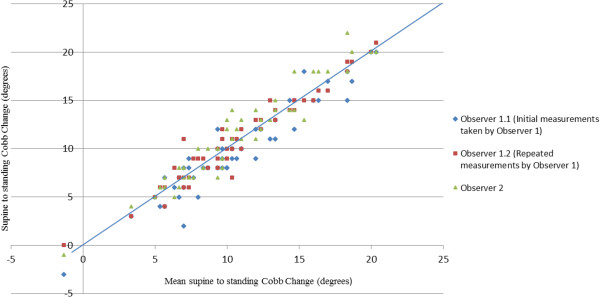
**Supine to standing Cobb change of each observation *versus *** **overall mean Cobb change for each patient.**

Where, *n* and *m* are the Cobb change measurements by the two observers. The mean absolute inter-observer difference was 2.1°, the SD of the difference was 1.9° and the 95% CI was ± 3.6°. There was no statistically significant correlation between inter-observer variability and standing Cobb measurements. Figure 7 shows a scatter plot of each observation of supine to standing Cobb change *versus* mean supine to standing Cobb change for each patient, with different symbols used for each observer. The mean supine to standing Cobb change includes both sets of measurements by Observer 1 as well as those by Observer 2.

## Discussion

The Cobb angle method is the most widely used technique for quantifying spinal curve severity and assessing scoliosis progression and treatment outcomes. While the majority of clinical assessments are performed on standing radiographs, supine imaging modalities such as CT or MRI are used in certain cases and can provide valuable additional information on the difference in scoliosis curve geometry between supine and standing positions. Knowing this difference also gives researchers and clinicians guidelines when interpreting supine imaging modalities (such as CT or MRI) where a standing (plane radiographic) measure may not be available. Whilst standing *versus* supine Cobb differences have been reported by several authors; to the best of our knowledge no previous studies have analysed the effect of endplate pre-selection on supine versus standing difference, nor have potential correlations between standing to supine Cobb change and patient characteristics been previously explored.

The primary aim of this paper was to determine whether endplate pre-selection affected the supine to standing Cobb angle change. The current study found that endplate pre-selection caused only a minor (less than 1°) increase in supine to standing Cobb change, and that the mean (11°) increase in supine to standing Cobb change reported in this study is broadly consistent with previous literature [2-5]. The position and the number of vertebrae comprising the major curve were also subject to change from supine to standing, although we note that most end vertebrae were constant between postures. Overall there was a small, clinically insignificant increase in the mean number of vertebrae in the major scoliotic curve from supine to standing. Taken together, these results suggest that it does not matter whether endplate pre-selection is used or not when measuring supine to standing Cobb change, as any effect of pre-selection on the results obtained will be less than observer measurement variability, and below the threshold of clinical significance for Cobb change. However, the magnitudes of Cobb change measured in this study confirmed that postural differences must be accounted for when comparing supine and standing images of spinal deformity patients.

The secondary aim of this paper was to identify whether any patient characteristics were linked with supine to standing Cobb change. Standing Cobb angle, age and fulcrum flexibility were all found to be statistically significant. In biomechanical terms, patients with larger standing Cobb angles have greater moments acting on their spine due to gravitational loading on the deformity, so it is intuitive that supine to standing Cobb change would be related to curve magnitude. It would also be expected that a stiffening of the major curve, related to age would reduce the supine to standing Cobb change. However one would also suspect that the curve may stiffen with skeletal maturity (Risser grade) but no statistically significant relationship between Risser grade and supine to standing Cobb change was found. This may be due to the uneven distribution of Risser grades for the entire cohort as; fourteen patients were classified Risser 0, three patients Risser 1, two patients Risser 2, seven patients Risser 3, fourteen patients Risser 4 and twelve patients Risser 5. Having said this, we believe there were an adequate number of patients in the Risser 0, 3, 4 and 5 groups to find a statistically significant correlation between supine to standing Cobb change and Risser had one been present.

The relationship of most interest is that between Cobb change and fulcrum flexibility. A current pre-operative clinical method for assessing spinal flexibility at our Center is the use of fulcrum bending radiographs. This method allows the clinician to estimate the curve correction which will be achievable with instrumented fusion surgery. The current study suggests that supine to standing Cobb angle change could be further investigated as an alternative flexibility measure for idiopathic scoliosis patients for cases where additional imaging is undesirable or not possible. With regard to the use of Cobb change as a measure of major curve flexibility, it is important to note the large range in Cobb changes, between -1° and +20° for the patient group. From a biomechanical perspective, the two measures are very different. The fulcrum flexibility method is a more specific measure that targets the apex of the major curve through local loading to predict curve correctability. A fulcrum is deliberately placed against the rib corresponding to the apex of the curve to reduce the effect of muscle activation. By contrast, the supine to standing Cobb change can be considered more as a globalized loading where factors such as increasing gravitational loading at lower vertebral levels and muscle activation could play a significant role. In addition to the coronal plane, the fulcrum bending and supine-to-standing flexibility measures are also likely to cause differences in the sagittal and transverse planes (particularly since some derotation of the rib hump is likely to occur during supine scanning). However, the primary focus of this present study was Cobb angle changes in the coronal plane as this is the plane of primary relevance in current clinical practice.

The combined R^2^ value for all three of the statistically significant variables in the Cobb change regression was only 0.38, suggesting that there may be other, as yet un-identified factors which affected supine to standing Cobb change. It is also possible that measurement variability reduced the coefficient of determination. The inter-observer and intra-observer measurement variability found in the present study is in agreement with reported (2.6° – 8.8°) ranges from previous studies [6,15-17], tending toward the lower range of measurement error. The 95% CI of ±3.1° for intra-observer variability was comparable to Shea *et al.*, who reported a 95% CI of ±3.3° error [18]. The 95% CI of ±3.6° for inter-observer variability was slightly lower than the 5-6° reported in the existing literature [19-21]. We note that in the present study, observer variability was assessed for Cobb *change* (i.e. the difference between a standing and supine Cobb angle), rather than for a single Cobb measurement as in previous variability studies.

## Conclusion

Pre-selection of vertebral endplates does not have a clinically significant effect on Cobb change between the supine and standing positions. The mean 11° supine to standing Cobb angle increase is consistent with previous literature. The number of vertebrae selected in the scoliotic major curve tended to increase from supine to standing, but was not clinically significant. Statistically significant correlations were found between supine to standing Cobb change and standing Cobb angle, age and fulcrum flexibility. Supine to standing Cobb change could be further investigated as a useful alternative measure of spinal flexibility in AIS patients.

## Competing interests

The authors declare they have no competing interests.

## Authors’ contributions

BK the corresponding author measured the Cobb angles, analysed the data and wrote the manuscript GA, RL and MI collected and maintained data and measured Cobb angles. CA assisted in data analysing and interpretation. MP, MI and CA reviewed and edited the manuscript. All authors read and approved the final manuscript.

## Supplementary Material

Additional file 1 Each patients mean supine Cobb angle with and without endplate pre-selection and standing Cobb angle. Click here for file
