# Supplementary material for: Supine to standing Cobb angle change in idiopathic scoliosis: the effect of endplate pre-selection
Source: Scoliosis. 2014 Oct 8;9:16. doi: 10.1186/1748-7161-9-16 (PMC4193912; doi:10.1186/1748-7161-9-16)
Supplement: Additional file 1 — Each patients mean supine Cobb angle with and without endplate pre-selection and standing Cobb angle. [file 1748-7161-9-16-S1.docx]

**Additional file 1**

Each patients mean supine Cobb angle with and without endplate pre-selection and standing Cobb angle.

| Patient number | Mean supine Cobb angle from CT **without** endplate pre-selection (°) | Mean supine Cobb angle from CT **with** endplate pre-selection(°) | Standing Cobb angle from clinical x-ray (°) |
| --- | --- | --- | --- |
| 1 | 46 | 46 | 54 |
| 2 | 38 | 36 | 45 |
| 3 | 37 | 35 | 42 |
| 4 | 33 | 33 | 45 |
| 5 | 55 | 55 | 62 |
| 6 | 45 | 42 | 63 |
| 7 | 38 | 37 | 48 |
| 8 | 39 | 40 | 49 |
| 9 | 35 | 34 | 44 |
| 10 | 48 | 48 | 54 |
| 11 | 42 | 42 | 53 |
| 12 | 47 | 45 | 58 |
| 13 | 41 | 40 | 55 |
| 14 | 30 | 30 | 50 |
| 15 | 44 | 40 | 52 |
| 16 | 43 | 42 | 50 |
| 17 | 50 | 51 | 60 |
| 18 | 48 | 47 | 60 |
| 19 | 42 | 42 | 52 |
| 20 | 40 | 36 | 47 |
| 21 | 40 | 40 | 53 |
| 22 | 55 | 55 | 54 |
| 23 | 36 | 36 | 42 |
| 24 | 38 | 32 | 48 |
| 25 | 39 | 38 | 52 |
| 26 | 48 | 48 | 58 |
| 27 | 32 | 30 | 40 |
| 28 | 45 | 45 | 60 |
| 29 | 39 | 39 | 59 |
| 30 | 46 | 50 | 64 |
| 31 | 31 | 30 | 43 |
| 32 | 36 | 35 | 48 |
| 33 | 49 | 50 | 68 |
| 34 | 39 | 39 | 42 |
| 35 | 40 | 40 | 58 |
| 36 | 35 | 38 | 52 |
| 37 | 28 | 28 | 38 |
| 38 | 30 | 28 | 46 |
| 39 | 35 | 39 | 48 |
| 40 | 37 | 35 | 48 |
| 41 | 41 | 43 | 56 |
| 42 | 43 | 41 | 50 |
| 43 | 32 | 31 | 42 |
| 44 | 47 | 44 | 53 |
| 45 | 47 | 44 | 56 |
| 46 | 45 | 40 | 52 |
| 47 | 41 | 41 | 48 |
| 48 | 42 | 43 | 58 |
| 49 | 42 | 40 | 57 |
| 50 | 46 | 44 | 55 |
| 51 | 43 | 43 | 52 |
| 52 | 51 | 51 | 56 |
